# DECISION-MAKING AND DISPARITIES IN QUALITY OF CHOSEN PLANS AMONG RACIAL-ETHNIC GROUPS IN MEDICARE ADVANTAGE

**DOI:** 10.1093/geroni/igz038.2535

**Published:** 2019-11-08

**Authors:** Maricruz Rivera-Hernandez, Kristy L Blackwood

**Affiliations:** 1 Brown University, Providence, Rhode Island, United States; 2 The Warren Alpert Medical School of Brown University, Providence, United States

## Abstract

Limited research regarding decision-making in Medicare Advantage (MA), which now disproportionally serves racial and ethnic minorities (~45% Hispanics and 30% African-Americans), has been conducted. Without understanding the extent to which vulnerable groups select low quality plans with high out-of-pocket costs (OOPC) and what factors influence this selection, these beneficiaries could continue to be adversely affected. The objective of this study is to understand plan choice decision-making process and differences in quality and OOPC of chosen plans between racial and ethnic minorities enrolled in MA. We used 2015 national data from Medicare and conducted in-depth interviews with 25 MA enrollees. African-Americans were enrolled in plans with higher drug deductibles and lower OOPC. In addition, Hispanics and African-Americans enrollment in high quality plans were lower by 10% and 5%, respectively, regardless whether mean OOPC were 100 or 1000 (P<.05). Our interviews highlighted issues regarding financial literacy, choice overload and complexity. These seniors did not use Plan Finder from Medicare and obtained information from insurance companies. Most seniors were confused about insurance terminology and expressed poor computer literacy. Among them, there was a prevailing sentiment that more expensive plans are better. Our findings could inform the Medicare program, and vulnerable populations who would benefit from plans that maximize quality of care with lower out-of-pocket spending. Finally, this information could contribute to state organizations’ future efforts such as ensuring quality of health services for older adults including. Overall, this research provides new evidence about an increasingly important part of our publicly funded health system.

